# Neuromedin U: A Small Peptide in the Big World of Cancer

**DOI:** 10.3390/cancers11091312

**Published:** 2019-09-05

**Authors:** Patrycja Przygodzka, Kamila Soboska, Ewelina Sochacka, Joanna Boncela

**Affiliations:** 1 Institute of Medical Biology, Polish Academy of Sciences, 106 Lodowa Str, 93-232 Lodz, Poland; 2 Faculty of Biology and Environmental Protection, University of Lodz, 141/143 Pomorska Str, 90-236 Lodz, Poland

**Keywords:** Neuromedin U, NMU, GPCR receptors, cancer

## Abstract

Neuromedin U (NMU), a neuropeptide isolated from porcine spinal cord and named because of its activity as a rat uterus smooth muscle contraction inducer, is emerging as a new player in the tumorigenesis and/or metastasis of many types of cancers. Expressed in a variety of tissues, NMU has been shown to possess many important activities in the central nervous system as well as on the periphery. Along with the main structural and functional features of NMU and its currently known receptors, we summarized a growing number of recently published data from different tissues and cells that associate NMU activity with cancer development and progression. We ask if, based on current reports, NMU can be included as a marker of these processes and/or considered as a therapeutic target.

## 1. Neuromedin U Structure: Implications for Biological Activity

Since it was identified in 1985, neuromedin U (NMU) expression has been found in many species in various isoforms. The longer forms NMU-17 (toad), NMU-21 (goldfish), NMU-23 (rat, tree frog), NMU-25 (human, pig, rabbit, dog, chicken, frog, goldfish), and the shorter forms NMU-8 (pig, dog) and NMU-9 (guinea pig, chicken) have been identified (reviewed in Reference [1]). Although there is still discussion about whether the longer forms of NMU intermediate precursors of the truncated forms are, there is agreement that the peptide is widely conserved between organisms, with emphasis on the amidated C-terminal pentapeptide (-Phe-Arg-Pro-Arg-Asn-NH2). The NMU sequence conservation indicates that its biological functions are tightly correlated with the peptide structure (reviewed in Reference [2]).

In addition to different NMU isoforms, Mori K et al. detected NMU-precursor-related peptides, NURP33 and NURP36, produced from the same NMU precursor in rat pituitary, small intestine, and brain tissues. The NURPs are involved in the pituitary release of prolactin, but they are unable to activate known NMU receptors [3]. Thus, the molecular mechanisms that controls various peptides’ synthesis from NMU precursors (alternative splicing, protease availability during peptide processing) seem to be vital for the regulation of their biological activity.

The picture is even more complicated, as a 36-amino-acid-long peptide, named neuromedin S (NMS), was also found in rat brain [1,4,5]. However, NMS is not a splicing variant of NMU. Although NMU and NMS have similar structures and receptor affinities and both primarily function as neuropeptides, NMU appears to be the focus of attention in the field of cancer development and progression.

Human neuromedin U is encoded by the *NMU* gene, located on chromosome 4 (4q12), and is synthesized as a 174-amino-acid precursor [6]. The first 34 aa at the N-terminus are the signal peptide, which is typical for the precursors of many other regulatory peptides. The mature NMU-25 sequence is located within the C-terminus of the pre–pro-peptide and is flanked by pairs of basic residues forming cleavage sites. The bioactivity of NMU from different species depends on two main features: a highly conserved pentapeptide at the C-terminus and post-translational amidation of the C-terminal amino acid, which is typical of many gastrointestinal hormones and determines receptor binding capacity [1]. Human pre–pro-NMU cleavage mainly generates NMU-25, but the presence of other putative proteolytic sites in the precursor suggests the possibility of releasing a series of other peptides, as shown in rats [3].

The distribution of NMU-25 in humans showed its expression in the central nervous system, gastrointestinal tract, oesophagus to rectum, genitourinary tract, thyroid gland, spleen, lymphocytes, adipose tissue, mast cells, endothelial cells, keratinocytes, and placenta [1].

## 2. Neuromedin U Receptors: Structure and Distribution

Neuromedin U plays its function through interaction with two main receptors: neuromedin U receptor 1 (NMUR1, previously FM-3, GPR-66) and neuromedin U receptor 2 (NMUR2, previously TGR-1), encoded by separate genes located on chromosomes 2 and 5, respectively. Both receptors share relatively high sequence and amino acid homology (~50%) [7,8] and demonstrate comparable sub-nanomolar affinity to NMU [9]. Both NMUR1 and NMUR2 were discovered as growth hormone secretagogue receptor (GHSR) and neurotensin receptor (NTSR) homologues, but testing of ghrelin, neurotensin, different types of neuromedins, and other similarly structured factors showed NMU and NMS as the only ligands for NMUR1 and NMUR2 [7,10,11,12,13]. Human receptor activation is, to some extent, NMU origin and isoform independent, as rat NMU-23, canine NMU-8, and porcine NMU-25 or NMU-8 induce signalling just as does human NMU-25 [9,11,12,14].

First reports identifying neuromedin U as a cognate ligand of orphan G-protein coupled receptors were published almost simultaneously by American and Japanese groups, and all distribution data were based on NMUR mRNA detection. The initial studies showed diversification in NMURs tissue distribution. NMUR1 was found to be prominently expressed in the periphery (e.g., gastrointestinal tract, male genitourinary system, lungs, kidneys, cardiovascular and immune system) and NMUR2 expression was mainly detected in the central nervous system [13]. Nonetheless, further studies complicated the picture and showed *NMUR1*’s presence in the cerebellum, hippocampus, and hypothalamus, while NMUR2 mRNA has been identified in peripheral tissues of genitourinary and gastrointestinal tracts and in many other organs [8,12,13]. This controversy emerged from the development of advanced detection techniques over recent years, and it can also be the effect of relatively high amino acid homology between NMUR1 and NMUR2, which implicates the shortage of effective experimental tools, such as highly specific antibodies. As antibody staining appeared to be non-specific and mainly inconsistent with RNA expression data (especially in the case of NMUR1, as seen in the Human Protein Atlas, among other sources), mRNA level is still used as the first measure when particular receptor presence is determined.

In addition to classical NMURs, Lin et. al. (2015) revealed a lack of the third exon in an *NMUR2* splice variant, *NMUR2S*, in human ovarian cancer. The expression of NMUR2S was also confirmed in various other human cancer cell lines [15].

Interestingly, the NMU receptor–ligand binding signal was also detected in cell lines without NMUR1 and NMUR2 expression. In non-small-cell lung cancer (NSCLC), Takahashi et al. (2006) found that the heterodimer formed by growth hormone secretagogue receptor 1b (GHSR1b) and neurotensin receptor 1 (NTSR1) was involved in NMU-related signalling [16]. Thus, it cannot be excluded that other NMU receptors could be found in the future.

All currently known NMU receptors NMUR1, NMUR2, NMUR2S and GHSR1b/NTSR1 belong to the large family of G-protein-coupled receptors (GPCRs). They have membrane localization and, except NMUR2S, classical GPCR structures with an extracellular N-terminus responsible for ligand binding, seven transmembrane domains, and an intracellular C-terminus [2,13,16]. Comparing two classical NMURs, NMUR2 has a shorter third intracellular domain as well as an N-terminus and a longer C-terminus than NMUR1 [2]. The truncated variant of NMUR2 (NMUR2S), due to the lack of the sixth transmembrane domain and the third extracellular loop, forms only six transmembrane domains with both the N- and C-termini, localized extracellularly [15], which is thought to be the major reason for NMUR2S’s negative modulation of NMU signalling.

### NMURs: Classical GPCRs

As previously mentioned, the approximately 50% amino acid homology between NMUR1 and NMUR2 implicates the shortage of effective tools to clearly define the functions of individual receptors. Many studies on the signal transduction triggered by NMUR activation have been performed with the use of cell lines with ectopic overexpression of particular NMUR and have been based on extensive knowledge of the GPCR receptor family [7,8,9,12,13,14,17].

**Figure 1:**
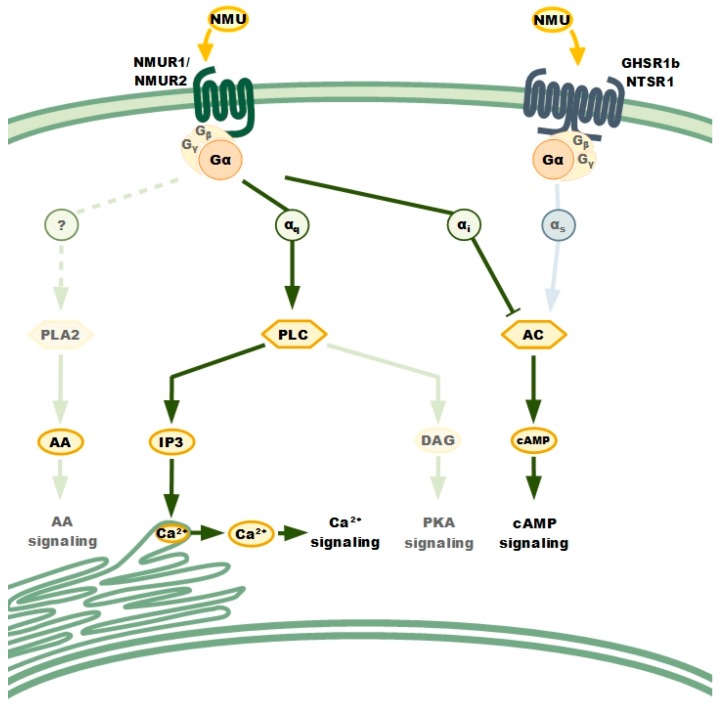
The current paradigm of neuromedin U signalling.

It is known that GPCRs propagate signals in the cell through heterotrimeric G-proteins. Upon ligand binding, GPCRs and G-proteins change their conformation and transduce signals inside the cell [18] (Figure 1).

Upon NMU binding, the receptor preferentially activates Gα_s_, Gα_i_, and Gα_q_ subunits. Stimulation of the Gα_s_ subunits activates adenyl cyclase (AC), whereas stimulation of the Gα_i_ subunits leads to its inhibition. Stimulation of the Gα_q_ subunits activates phospholipase C (PLC). NMURs dimerization, homo- or heterodimerization is implicated in their activation as well as signalling. The analysis of NMURs’ signal pathway was based on functional study comprising cancer cells lines with individual receptor overexpression and using Pertussis toxin (PTX). Phospholipase A2 (PLA2), Aarachidonic acid (AA), inositol 1,4,5-trisphosphate (IP3), diacylglycerol (DAG), cAMP-dependent protein kinase (PKA).

Both activated NMUR1 and NMUR2 regulate signalling pathways involving inositol phosphates and calcium as secondary messengers [8,9,12,17]. Studies based on the various sensitivities of different G-protein subunits to the pertussis toxin (PTX; Gα_q_ insensitive, Gα_i_ sensitive) have established which subunit of G-proteins are involved in NMURs signal transduction. PTX pre-treatment of HEK-293 or COS-7 or CHO cells overexpressing either NMUR1 or NMUR2 did not interfere with calcium mobilization after NMU application, indicating that Gα_q_ is the main player in the process [7,8,9,12,13].

Subsequently, phospholipase C was identified as an enzyme underlying phosphoinositide metabolism and the calcium mobilization response [7,8,9,12,13]. Finally, the application of aminosteroid U-73122 (an inhibitor of phospholipase C), which abolished NMU-stimulated inositide phosphate formation, confirmed the involvement of phospholipase C in NMURs signalling [9].

Besides the identification of G-proteins and the analysis of effector proteins engaged in NMU signalling, few downstream processes have been studied. Following calcium mobilization dynamics, it was found that the initial Ca^2+^ arises from the sarco-/endoplasmic reticulum, but interestingly, it is sustained by a transmembrane calcium gradient [17]. Arachidonic acid metabolites are released upon NMU treatment and are assumed to be a result of calcium signalling and calcium-dependent activation of phospholipase A_2_ [7,14].

Other pathways typical of GPCR signalling analysed in the scope of NMUR signal transduction involve modulation of adenylate cyclase activity and cAMP production. The NMU interference with forskolin-stimulated increases in cAMP was shown to depend on the cellular background and/or experimental settings used. The activation of transiently expressed NMUR1 in HEK293 cells had no effect on the cAMP level, which excluded the contribution of the Gα_i_ and Gα_s_ subunits to NMUR1 signalling [12]. Nevertheless, NMU partially inhibited forskolin-stimulated cAMP accumulation in a CHO cell line with stable expression of NMUR2 [7]. Several further studies using HEK-293 clones overexpressing either NMUR1 or NMUR2 confirmed the suppression of forskolin-mediated cAMP accumulation after NMU application, thereby indicating the functional relevance of Gα_i_ activation [9,17]. Interestingly, inhibition of cAMP accumulation was more pronounced in NMUR2-expressing cells than in NMUR1-expressing cells, which seems to be related to the different affinities of NMUR1 and NMUR2 to various Gα subunit types [9]. Additional studies with PLC inhibitors revealed partial abolishment of NMUR2-activated inhibition of cAMP accumulation, which indicates the participation of the PLC signalling pathway in the regulation of cellular cAMP level [9].

GPCR-mediated activation of the MAPK pathway is mechanistically complex but well documented, and NMURs are not the exception in this family. Signal transduction mediated by NMU through NMURs can increase ERK phosphorylation in 5–10 min. ERK phosphorylation follows both receptors’ activation and is insensitive to pertussis toxin [17,19]. Nonetheless, the precise signal from activated NMURs to ERK phosphorylation is still undetermined.

NMURs availability for ligands and further signal transduction is also regulated by receptor recycling processes common to other GPCRs [19]. Willars’s group showed that NMU binds irreversibly to NMURs under physiological conditions, leading to a decreased cell response to NMU treatment, NMURs desensitization, and internalization [17,19].

Little is known about the regulation of signalling pathways mediated by the other NMU receptors.

Lin et al. confirmed the lack of NMU-dependent signalling in cancer cells overexpressing NMUR2S and showed the role of the truncated receptor in the negative regulation of NMU activity by forming heterodimers with NMUR1 or NMUR2. One possible explanation of this phenomenon is the decreased NMUR2S potential for binding NMU and its reduced ability to trigger signalling pathways, probably as the effect of receptor structure in which both the N- and C-termini are localized extracellularly [15].

The heterodimer NTSR1/GHSR1b, which was found to pass down the NMU signal in lung cancer cells, intensified the production of cAMP but not intracellular calcium mobilization. It was hypothesized that the NTSR1/GHSR1b signalling pathway varies from classical NMURs action and is mediated by the Gα_S_ subunit. NMU binding to NTSR1/GHSR1b may lead to activation of adenylyl cyclase to increase cAMP and subsequent activation of PKA and transcription factors involved in control of cancer cell growth promotion [16].

Despite all these data, there are still many questions concerning the NMU receptors, as most of the observations have not yet been shown on endogenously expressed NMURs, and its physiological relevance still needs to be revealed [17].

## 3. NMU in Cancer: Knowing the Future Through the Present

NMU has been associated with a myriad of different functions (reviewed in Reference [20]), from the regulation of eating behaviour, energy homeostasis, blood pressure, and muscle contraction to pain perception, prolactin secretion, and sleep regulation. NMU knockout mice studies showed that mice develop obesity and NMU plays an important role in the regulation of feeding behaviour and energy metabolism independent of the leptin signalling pathway [21]. In various types of cancer, NMU expression was identified by high-throughput analysis, such as tissue microarray analysis or gene expression profiling of patient samples. These observations were enriched by various analyses and collation of data from public databases such as The Cancer Genome Atlas (TCGA) (https://tcga-data.nci.nih.gov/), Gene Expression Omnibus (GEO) (http://www.ncbi.nlm.nih.gov/geo/) and The Human Protein Atlas (THPA) (www.proteinatlas.org) [22] that enabled the correlation of NMU expression with cancer stage and patient survival.

So far, the reported findings connecting NMU with cancer have been ambiguous and dependent on cancer type and stage as well as the experimental model (Table 1). Nevertheless, the small secretory peptide with a very low concentration in plasma (biological half-life in the blood—4 min) and behaviour of locally acting molecule rather than a circulating hormone [1] have become a point of interest in the field of cancer studies. 

### 3.1. NMU as A Tumour Suppressor

The first report that linked neuromedin U to cancer showed *NMU* was significantly downregulated in each of the five examined oral squamous cell carcinoma (OSCC) samples compared to healthy tissues [39]. Other studies revealed that the *NMU*-promoter region was hypermethylated in 3 out of 10 oesophageal squamous cell carcinoma (ESCC) primary tumours tested, while in healthy tissues, this phenomenon was not observed [23]. Hypermethylation correlated with a decrease in NMU expression level, suggesting that its silencing was associated with its tumour-suppressive activity. The *NMU* promoter methylation in a tumour-specific manner was also reported in 20% of head and neck squamous cell carcinoma (HNSCC) samples [24], but recently published data on HNSCC [25] showed NMU upregulation in the advanced stage of this tumour. A proposed *NMU* function as a tumour suppressor gene was not further investigated.

### 3.2. NMU as A Prognostic Factor

Interestingly, after initial reports implying the suppressive effects of NMU in oesophageal and head and neck squamous-cell carcinomas, many other studies demonstrated neuromedin U as a diagnostic and/or prognostic biomarker in various tumours.

The analysis of lung cancer tissue samples from non-small-cell lung carcinoma (NSCLC, stage I to III) and small-cell lung carcinoma (SCLC, stage IV) showed NMU overexpression compared to normal lung tissue. In the cDNA microarray, NMU transcript was identified to be frequently overexpressed (5 fold higher expression) in the majority of NSCLC cases (over 50%). Western blot analysis confirmed the increased expression of NMU protein in lung cancer tissue, and the results were consistent with RT-PCR data. The NMU-positive staining was observed in 68% of NSCLC and 82% of SCLC samples. The immunohistochemical analysis of tissue microarrays, including 326 

NSCLS tissues, demonstrated GHSR1b-positive staining in 218 cases, and 217 cases were positive for NTSR1. The expression pattern of GHSR1b and NTSR1 was significantly correlated with the NMU expression pattern in the examined lung cancer tissue samples. Moreover, patients with NSCLC and NMU-positive tumour staining showed significantly shorter cancer-specific survival times than patients with NMU-negative tumours (*p* = 0.036) [16].

Similar observations have been made in breast cancer, where *NMU* has been proposed as a prognostic biomarker for poor outcome, mainly in HER2-overexpressing tumours [34,36]. A negative correlation between *NMU* level and overall survival was shown in groups with HER2-positive, advanced, large, triple-negative and luminal A tumour subtypes [34,36]. Interestingly, Garczyk S et al. recently showed a correlation between *NMU* mRNA expression in breast tumours and poor prognosis, but only in patients with high *NMUR2* expression. For the other tested receptors (*NMUR1*, *NTSR1*, *GHSR*), no similar observations were made [34], suggesting the dominant role of NMUR2 in NMU signalling. The immunohistochemical staining of breast tumours identified the NMU protein, but only in those tissues that showed very high expression of *NMU* mRNA.

Clear cell renal cell carcinoma (CCRCC) is another cancer where an increase in the NMU level was revealed in the analysis of publicly available microarray databases comparing normal kidney and tumour tissue from patients with sporadic CCRCCs [37]. These observations were confirmed by subsequent analysis of newer data comprising transcript and protein levels generated by the TCGA Research Network and the THPA database, where NMU was defined as an unfavourable prognostic marker in renal cancer (www.proteinatlas.org) [22].

Likewise, two studies combining data concerning endometrial carcinoma RNA sequencing with data downloaded from TCGA and GEO databases indicated an increase in NMU expression in all types and grades of the primary tumours compared to normal endometrial tissues [32,33]. These results were further confirmed in the endometrial tissue microarray analysis, where the NMU protein staining signal was elevated in cancer samples. Patients with high *NMU* levels had serious effects of low overall survival and low recurrence-free survival [32], which was also validated by the THPA, which defined NMU as a marker for poor outcome of endometrial cancer (www.proteinatlas.org) [22].

Validation of NMU, NMUR1, and NMUR2 levels in samples of patients with ovarian cancer and in paired normal adjacent tissues (*n* = 100) showed that in this type of tumour, the level of *NMU* expression was elevated (941 fold). Moreover, the *NMUR2* expression level was increased (5 fold). Immunohistochemical staining confirmed the observed transcript changes [15].

The list of tumours with a significant increase in the level of *NMU* is extended by pancreatic adenocarcinoma [26], whereas in the healthy pancreas from organ donors and in chronic pancreatitis, the *NMU* level was very low, almost undetectable. The *NMUR1* expression was found at the same low level in all pancreatic tissues, but the amount of *NMUR2* mRNA detected in pancreatic cancer tissues was 149 times higher compared to healthy tissues. The obtained results were confirmed by protein analysis. The immunohistochemical staining showed neither NMU nor NMUR2 immunoreactivity in the normal pancreas, while in the pancreatic cancer tissues, there was very high cytoplasmic staining of NMU and NMUR2, which was also detected in the cell membrane. Interestingly, NMU serum levels decreased after tumour resection [26].

The most recent reports added papillary thyroid carcinoma (PTC) to a group of cancers with high *NMU* expression correlated with decreased disease-free survival time, as shown by an analysis of two independent GEO datasets with bioinformatics tools [38].

All these findings suggest a critical contribution of NMU to cancer growth and development and support the hypothesis that NMU serves as a marker of poor prognosis and short patient survival [16,25,32,34].

### 3.3. Effect of NMU on Metastasis Formation

In addition to NMU abundance in cancer tissues, significant shifts in peptide expression were also reported in metastatic conditions, suggesting a possible contribution of NMU not only to cancer development and growth but also to cancer progression.

The abovementioned studies on squamous-cell carcinoma [25] ended the initial perception of NMU as a potential cancer suppressor but also for the first time suggested NMU as a biomarker of regional metastasis. The immunostaining of the samples from patients histologically diagnosed with HNSCC, as well as the immunostaining of commercially available tissue microarrays, consisted of head and neck cancers, tongue, laryngeal and nasal carcinoma revealed the predominant NMU expression in the primary tumours with metastasis compared to the primary tumours without metastasis [25].

In breast cancer, a significant negative correlation was also noted between NMU expression level in primary tumours and overall survival rate in nodal positive patients [34].

Pancreatic cancer studies have provided to date the sole observations of *NMU* expression increases in metastatic tissues of the liver and lymph nodes [26]. Moreover, *NMUR1* levels were also elevated in the metastatic tissues compared to their healthy counterparts. Considering that the current results regarding NMU receptor signalling [16] do not discriminate between NMUR1 and NMUR2 signal transduction, it is interesting that metastatic tissues express more NMUR1 but pancreatic primary tumours “invest” in NMUR2 overproduction.

More data concerning the role of NMU in metastasis were acquired from molecular studies of cancer cells described during the course of the review.

## 4. NMU Signalling in Cancer Cells Biology

Since detailed, functional studies are not possible in patient samples, much effort has been put into experiments that employed cell line models. Recombinant NMU treatment, ectopic overexpression or silencing of NMU and its receptors in cancer cell lines were used to clarify the function of NMU in different cellular processes. Here, we summarize current knowledge and collected facts about NMU activity at the cellular level (Table 1).

### 4.1. Cancer Cells Proliferation and Viability

As NMU appears to be a promising diagnostic marker in various cancer types, researchers have tried to elucidate its role in cancer cell proliferation and survival, but the results appeared to be cancer-type dependent.

In squamous cell carcinoma cell lines, a suppressive role of NMU related to hypermethylation of its promoter has been identified, as was observed in tissues [23,24]. NMU showed a marked reduction in expression or was shown to be completely silenced in 12 examined ESCC cell lines. Functional experiments on KYSE30 cells demonstrated diminished colony formation efficacy after NMU treatment [23]. The authors concluded that the peptide has potent growth-suppressive activity. 

In contrast, it was shown that NMU together with NMUR1 promotes the growth of human myeloid leukaemia cells. This was found by microarray analysis of K562 cells with dominant-negative Myb proto-oncogene (MERT) expression. The transcription factor c-Myb regulates a unique set of genes in leukemic cells that are required for survival. NMU was identified as a c-Myb target gene upregulated in human leukaemia cells that stimulates colony formation. This relationship was confirmed in primary acute myelogenous leukaemia cells (AML), where NMU supplementation resulted in increased cell proliferation in 3 out of 4 examined patient-derived cell lines. Moreover, silencing *NMU* expression decreased AML cell viability [27].

Studies of endometrial cancer cells reflect tissue observations. The increased level of NMU expression in endometrial cancer cells positively correlated with cell proliferation. Functional studies of cells derived from patients with endometrial tumours of grade II showed that NMU signalling promoted cell proliferation, whereas NMU knockdown caused cell growth inhibition as the effect of cell cycle arrest, but not cell death [32,33].

Neither repressing nor promoting effects of NMU on cell proliferation were observed in renal cancer cells (RCC10/VHL cell line) [37] nor in pancreatic cancer cell lines (Capan1, SU86.86, MiaPaca2, Panc1) [26].

### 4.2. EMT, Cancer Cell Motility, and Invasiveness

In many types of cancer, new therapies improve the overall survival, but once metastasis becomes clinically apparent, the prognosis becomes poor and survival is shortened. One of the first steps of local dissemination from solid tumours and the subsequent evolution of metastasis is the epithelial–mesenchymal transition programme (EMT). Transformation of epithelial carcinoma into mesenchymal-like, more motile and invasive cells is the core process that facilitates the escape of cancer cells from their primary location. A few observations at the cancer tissue level described above are supported by a relevant number of reports from cancer cell experiments concerning NMU’s association with metastasis.

A substantial contribution to the field of NMU involvement in breast cancer was made by the O’Driscoll group [35,36], who correlated NMU with HER2-positive cells’ (HCC1954 and SKBR3 cells) therapy resistance and elevated aggressiveness, evinced by increased migration, invasion, and resistance to anoikis, whereas NMU knockdown had the opposite effects [36]. Further studies showed that NMU overexpression or treatment led to upregulation of EMT markers and increased secretion of IL-6 by breast cancer cells, which together with the cellular metabolic switch from preferential use of mitochondrial respiration to glycolysis suggested that NMU enhances drug resistance by conferring a cancer stem cell (CSC) phenotype. Additionally, NMU-induced IL-6 secretion was proposed as one of the mechanisms through which NMU increases cell migration [35]. Other studies confirmed that NMU promotes a motile phenotype and showed its growth-inhibitory effect in NMUR2-positive (SKBR3) but not NMUR2-negative (Hs578T) breast cancer cells, indicating the importance of NMUR2-related signalling [34]. Transcription analysis of SKBR3 cells stably overexpressed NMU allowed the establishment of NMU-related gene signatures. Several cancer-relevant pathways appear to be significantly affected, i.e., Wnt, Ephrin receptor, TGFB, and ERK. Since the crosstalk between Wnt and NMU signalling was previously shown, these pathway components were extensively analysed. The decreased expression of the canonical Wnt target MYC and enhanced activation of the non-canonical Wnt/planar cell polarity (PCP) pathway effector RAC1 were found and validated. These components were suggested as signal contributors to growth inhibition and promotion of cell migration in breast cancer cells overexpressing NMU.

The co-expression of NMU and NMUR2, validated at the mRNA and protein levels, was also correlated with increased invasiveness and metastatic potential in pancreatic cancer cells (i.e., ASPC1, Capan1, Colo357, SU86.86, BxPC3, Capan1). Microarray assays and qRT-PCR analysis of cells treated with NMU identified several potential target genes. Among them, the c-Met oncogene is genetically altered or overexpressed in many human cancers. The authors reported that NMU, by inducing c-Met, increased cell motility and invasiveness as well as hepatocyte growth factor (HGF)-induced scattering of pancreatic cancer cells [26].

Signalling induced by NMU binding to NMUR2 also appears to be crucial in endometrial cancer cells. Functional studies have shown that NMU signalling promotes the motility of cells isolated from grade II tumours. Further investigations explained how NMU signalling may contribute to EGF- or TGFB-induced EMT through elevated expression of adhesion molecules, such as CD44 and integrins and their corresponding ECM ligands, hyaluronan and collagen IV, as well as through increased SRC kinase activity and its downstream effectors’ actions, GTPases, RAC1, and RhoA. Moreover, NMU regulation was shown to be a potential target of HAND2-AS1, lncRNA, and tumour suppressors in ECC [32,33].

The relationship of NMU with transcription factors was also found in lung cancer cells. Microarray analysis of LC319 cells with silenced *NMU* indicated that the FOXM1 transcription factor is a downstream target in the NMU signalling pathway. Observations from cell and tissue studies suggest that the NMU-FOXM1 pathway promotes the malignant nature of lung cancer cells [16].

The NMU status of unfavourable markers in renal cancer was examined in cancer cells in relation to VHL (von Hippel-Lindau) protein. Inactivation of this tumour suppressor is detected in most clear cell renal cancers. NMU expression was tested in two *VHL*-defective renal cancer cell lines (i.e., RCC4 and RCC10) and their sublines with stable overexpression of VHL. The obtained results showed that *NMU* expression was markedly increased in the absence of functioning VHL. Further investigation demonstrated that the increase in *NMU* expression was dependent on the activation of hypoxia-inducible factor (HIF). Unlike previously described cancers, renal cancer mainly expresses NMUR1, whose functional activity was confirmed. Ectopic expression of NMU in RCC10/VHL cells was found to significantly enhance their migration and invasion abilities [37].

In the case of two cancer types, colon and bladder cancer, there are no data on NMU tissue expression level, and all published data are from cell culture studies.

In our investigations of colon cancer cells (HT29), we found that NMU and NMUR2 are co-expressed in colon cancer cell lines with induced EMT. Our data associate NMU expression and release with colon cancer progression and Snail transcription factor activity, a key EMT inducer [30].

In bladder cancer, NMU was found to be regulated by the lung metastasis suppressor RhoGDI2 (GDP dissociation inhibitor 2) [28,29]. The signalling pathway remains unknown, but *NMU* expression was shown to be downregulated in the tumorigenic and metastatic cell line T24T as a result of RhoGDI2 reconstitution. The overexpression of NMU in T24T promoted anchorage-independent growth, but no effect was detected in the non-metastatic counterpart cell line T24. Interestingly, elevated levels of NMU promoted in vivo tumorigenicity. The authors suggested that NMU’s impact on tumour formation is possibly strongly related to the tumour microenvironment. NMU enhanced pulmonary metastasis, but only in cells with more metastatic features (T24T), implying that the peptide alone is insufficient to spark the metastatic cascade [28,29].

### 4.3. NMU’s Contribution to Cancer Cell Drug Resistance

Drug resistance is a well-known phenomenon in cancer [40]. The insensitivity of cancer cells to therapeutic agents is considered to be the main cause of failure of therapy and mortality of patients. Despite the initial positive reaction to chemotherapeutics, resistance often develops due to the various changes occurring in and outside cancer cells.

In breast and lung cancers, it has been proven that NMU may be an important resistance-enhancing factor. Rani et al. [36] studied a panel of breast HER-positive cell lines (SKBR3, HCC1954, MDA-MB-351, T47D) sensitive or resistant to HER-targeting drugs (lapatinib, trastuzumab, neratinib and afatinib). The obtained results showed the increased expression and secretion of *NMU* mRNA from all tested resistant cell lines. This observation indicated that the elevated expression of *NMU* is a part of cancer cells’ early response to short-term drug exposure. In addition, studies have shown that the level of NMU expression can impact on drug sensitivity. NMU overexpression in sensitive cells conferred resistance to the tested drugs, whereas NMU silencing sensitized resistant cells [36]. Further experiments indicated that heat shock protein 27 (HSP27), as a binding partner for NMU, conferred drug resistance. HSP27 was shown to stabilize HER2 and, thus, increase cell resistance to HER-targeted drugs. The recent integrative bioinformatic analysis of NSCLC samples showed *NMU* is potentially involved in conferring resistance to alectinib by interacting with five genes previously shown to be involved in conferring drug resistance, i.e., *CD80*, checkpoint kinase 1 (*CHEK1*), MYCN proto-oncogene, bHLH transcription factor (*MYCN*), pim-1 proto-oncogene (*PIM1*), and interleukin 1 receptor antagonist (*IL1RN*). Annotation of the biological processes of *NMU* and drug resistance in NSCLC indicated that NMU may confer alectinib resistance via multiple mechanisms, e.g., affecting cell viability, cell cycle, adhesion, and migration [31]. The results described by Rani et al. [36] and by You and Gao [31] suggest the involvement of NMU in the drug resistance process. Thus, silencing NMU can be an effective way to restore the sensitivity of cancer cells.

## 5. Future Perspectives

A growing number of reports draw our attention to the feasible tumorigenic and pro-invasive action of NMU (Table 1) but defining the upstream and downstream processes and signalling pathways that could become a therapeutic target remains challenging.

In addition to NMU protein secretion, *NMU* mRNA was found in extracellular vesicles released from CRC cells [30]. Moreover, it has to be remembered that NMU is released not only from cancer cells. Thus, its autocrine and paracrine effect on both cancer cells and tumour niche cells is expected but not explored. NMU receptors are expressed on, among others, macrophages and endothelial cells, known to be active modulators of the tumour microenvironment. In bladder cancer, NMU increased tumour formation and metastasis only in vivo [29], indicating that the impact of NMU is highly related to the tumour microenvironment.

As NMU itself displays an anorexigenic effect in animals and alters cancer cell bioenergetics, deeper insight into NMU as a cancer cachexia regulator would be interesting. The preliminary work carried out on bladder cancer in this regard seems to be insufficient, since the attenuation or reversal of cancer cachexia is very difficult once it appears.

**Figure 2:**
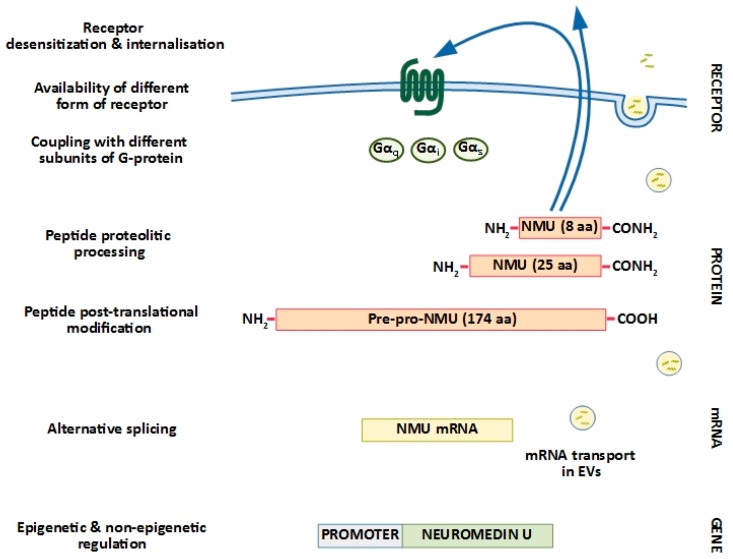
NMU expression and activity is regulated at multiple cellular levels.

In conclusion, NMU is already considered a tumour growth and/or progression marker in endometrial, renal, and breast cancers. Nevertheless, more basic research is needed to reveal its role in other cancer types and in the cancer microenvironment before it can be proposed as a therapeutic target. The molecular mechanisms impacting on peptide synthesis and activity appear to encompass multiple levels. Thus, it is becoming apparent that the overall integration of genetic, epigenetic, transcriptional and post-transcriptional mechanisms of regulation is the critical factor of whether NMU is able to fulfil its function (Figure 2). The work ahead leading to its full recognition and characterization in cancer cells will certainly contribute to a broader understanding of NMU function in cancer. 

## Figures and Tables

**Table 1:** Cell signalling related to NMU in various cancers. Grey charts present factors which influence NMU expression and white charts factors affected by NMU expression in cancer.

Cancer Type	Expression of *NMU* in Tissues (Cancer/Healthy)	NMU Receptors		Signal Contributors	Observed Biological Effects
		Expression	Signal Transduction Research		
Oesophageal [23]	↓	no data	no data	no data	*NMU* silencing in cells and cancer tissue is a result of *NMU*-promoter region hypermethylation. NMU treatment caused diminished colony formation efficacy of cancer cells.
Head and neck [24,25]	↑ [24]	no data	no data	no data	*NMU*-promoter region hypermethylation [25]. NMU upregulation in the advanced stage of cancer [24].
Pancreatic [26]	↑	NMUR2	no data	c-Met	NMU and NMUR2 upregulation in the cancer tissues and cancer cell lines correlates with increased invasiveness and metastatic potential of cells. *NMU* and *NMUR2* expression upregulation in metastatic tissues of the liver and lymph nodes.
Leukaemia [27]	no data	NMUR1	NMUR1	c-Myb	NMU treatment resulted in the increased leukaemia cells proliferation and increase in colony formation ability. *NMU* silencing resulted in decrease in leukaemia cells viability.
Bladder [28,29]	no data	no data	no data	RhoGDI2	NMU expression in cells with metastatic features enhanced pulmonary metastasis
Colorectal [30]	↑	NMUR2	no data	Snail	NMU upregulation in cancer cells at the early stage of EMT. *NMU* mRNA detected in microvesicle fraction released from invasive cells.
Lung [16,31]	↑ [16]	GHSR1b/NSTR1 (heterodimer)	GSHR1b/NSTR1	FOXM1 [31] CD80 [31] CHEK1 [31] IL1RN [31] MYCN [31] PIM1 [31]	NMU upregulation in the cancer tissues and cell lines led to increase in cancer cells growth and invasion [16]. NMU is potentially involved in cancer cells resistance to alectinib [31]. *NMU* silencing resulted in decreased cells viability and ability to form colonies [16].
Endometrial [32,33]	↑ [32,33]	NMUR1 (low) NMUR2 (high)	NMUR2	ITGA1 [32] CD44 [32] MMP-2,3,9 [32] COLA4A1 [32] COLA4A2 [32] HAS3 [32] HYAL1 [32] HYAL2 [32] HYAL3 [32] c-SRC [32] RAC1 [32] RHOA [32] TGFB [32] EGF [32] HAND2-AS1 [33]	NMU upregulation in the cancer tissues correlated with poor outcome. NMU upregulation in cell lines increased cell proliferation and motility of cells isolated from grade II tumours [32]. *NMU* silencing resulted in decreased cells migration, invasion, proliferation, and adhesion [32,33]. Cancer cells with decreased NMU expression formed smaller tumours in mice models [32].
Breast [34,35,36]	↑ [34]	NMUR1 NMUR2 NSTR1	NMUR1 [36] NMUR2 [34,36]	WNT (Myc, RAC1) β-catenin [35] E-cadherin vimentin [35] TGFB Ephrin receptor	NMU upregulation in the cancer tissues was proposed as prognostic biomarker for poor outcome [34,36]. NMU upregulation on the cellular level caused [34,35,36]: Decrease in colony formation ability and viability Increase in migration, invasion, motility. and resistance to anoikis Switch to glycolysis Increase in secretion of IL-6 Increase in EMT markers expression Expansion in CSC phenotype Resistance to lepatynib, trastuzumab, peratynib, and afatynib
Renal [37]	↑	NMU1R (low)	no data	VHL HIF-1α HIF-2α	NMU upregulation in the cancer tissues was proposed as prognostic biomarker for poor outcome. NMU upregulation on the cellular level caused increased migration and invasion ability.
Ovary [15]	↑	NMUR1 NMUR2 NMUR2S	NMUR1 NMUR2	no data	*NMU* and *NMUR2* expression was increased in cancer tissues and on the cellular level significantly enhanced migration and invasion abilities.
Thyroid [38]	↑	no data	no data	no data	*NMU* upregulation correlated with patients decreased disease-free survival time.
Oral [39]	↓	no data	no data	no data	no data

## References

[1] Mitchell J.D., Maguire J.J., Davenport A.P. (2009). Emerging pharmacology and physiology of neuromedin U and the structurally related peptide neuromedin S. Br. J. Pharmacol..

[2] Brighton P.J., Szekeres P.G., Willars G.B. (2004). Neuromedin U and its receptors: Structure, function, and physiological roles. Pharmacol. Rev..

[3] Mori K., Ida T., Fudetani M., Mori M., Kaiya H., Hino J., Nakahara K., Murakami N., Miyazato M., Kangawa K. (2017). Identification of neuromedin U precursor-related peptide and its possible role in the regulation of prolactin release. Sci. Rep..

[4] Mori K., Miyazato M., Ida T., Murakami N., Serino R., Ueta Y., Kojima M., Kangawa K. (2005). Identification of neuromedin S and its possible role in the mammalian circadian oscillator system. EMBO J..

[5] Mori K., Miyazato M., Kangawa K. (2008). Neuromedin S: Discovery and functions. Results Probl. Cell Differ..

[6] Austin C., Lo G., Nandha K.A., Meleagros L., Bloom S.R. (1995). Cloning and characterization of the cDNA encoding the human neuromedin U (NmU) precursor: NmU expression in the human gastrointestinal tract. J. Mol. Endocrinol..

[7] Hosoya M., Moriya T., Kawamata Y., Ohkubo S., Fujii R., Matsui H., Shintani Y., Fukusumi S., Habata Y., Hinuma S. (2000). Identification and functional characterization of a novel subtype of neuromedin U receptor. J. Biol. Chem..

[8] Raddatz R., Wilson A.E., Artymyshyn R., Bonini J.A., Borowsky B., Boteju L.W., Zhou S.Q., Kouranova E.V., Nagorny R., Guevarra M.S. (2000). Identification and characterization of two neuromedin U receptors differentially expressed in peripheral tissues and the central nervous system. J. Biol. Chem..

[9] Aiyar N., Disa J., Foley J.J., Buckley P.T., Wixted W.E., Pullen M., Shabon U., Dul E., Szekeres P.G. (2004). Radioligand binding and functional characterization of recombinant human NmU1 and NmU2 receptors stably expressed in clonal human embryonic kidney-293 cells. Pharmacology.

[10] Tan C.P., McKee K.K., Liu Q.Y., Palyha O.C., Feighner S.D., Hreniuk D.L., Smith R.G., Howard A.D. (1998). Cloning and characterization of a human and murine T-cell orphan G-protein-coupled receptor similar to the growth hormone secretagogue and neurotensin receptors. Genomics.

[11] Kojima M., Haruno R., Nakazato M., Date Y., Murakami N., Hanada R., Matsuo H., Kangawa K. (2000). Purification and identification of neuromedin U as an endogenous ligand for an orphan receptor GPR66 (FM3). Biochem. Biophys. Res. Commun..

[12] Szekeres P.G., Muir A.I., Spinage L.D., Miller J.E., Butler S.I., Smith A., Rennie G.I., Murdock P.R., Fitzgerald L.R., Wu H.L. (2000). Neuromedin U is a potent agonist at the orphan G protein-coupled receptor FM3. J. Biol. Chem..

[13] Shan L.X., Qiao X.D., Crona J.H., Behan J., Wang S., Laz T., Bayne M., Gustafson E.L., Monsma F.J., Hedrick J.A. (2000). Identification of a novel neuromedin U receptor subtype expressed in the central nervous system. J. Biol. Chem..

[14] Fujii R., Hosoya M., Fukusumi S., Kawamata Y., Habata Y., Hinuma S., Onda H., Nishimura O., Fujino M. (2000). Identification of neuromedin U as the cognate ligand of the orphan G protein-coupled receptor FM-3. J. Biol. Chem..

[15] Lin T.Y., Huang W.L., Lee W.Y., Luo C.W. (2015). Identifying a Neuromedin U Receptor 2 Splice Variant and Determining its Roles in the Regulation of Signaling and Tumorigenesis in vitro. PLoS ONE.

[16] Takahashi K., Furukawa C., Takano A., Ishikawa N., Kato T., Hayama S., Suzuki C., Yasui W., Inai K., Sone S. (2006). The neuromedin U-growth hormone secretagogue receptor 1b/neurotensin receptor 1 oncogenic signaling pathway as a therapeutic target for lung cancer. Cancer Res..

[17] Brighton P.J., Szekeres P.G., Wise A., Willars G.B. (2004). Signaling and ligand binding by recombinant neuromedin U receptors: Evidence for dual coupling to G alpha(q/11) and G alpha(i) and an irreversible ligand-receptor interaction. Mol. Pharmacol..

[18] Cabrera-Vera T.M., Vanhauwe J., Thomas T.O., Medkova M., Preininger A., Mazzoni M.R., Hamm H.E. (2003). Insights into G protein structure, function, and regulation. Endocr. Rev..

[19] Alhosaini K., Bahattab O., Qassam H., Challiss R.A.J., Willars G.B. (2018). Ligand-Specific Signaling Profiles and Resensitization Mechanisms of the Neuromedin U_2_ Receptor. Mol. Pharmacol..

[20] Martinez V.G., O’Driscoll L. (2015). Neuromedin U: A Multifunctional Neuropeptide with Pleiotropic Roles. Clin. Chem..

[21] Hanada R., Teranishi H., Pearson J.T., Kurokawa M., Hosoda H., Fukushima N., Fukue Y., Serino R., Fujihara H., Ueta Y. (2004). Neuromedin U has a novel anorexigenic effect independent of the leptin signaling pathway. Nat. Med..

[22] Uhlen M., Fagerberg L., Hallstrom B.M., Lindskog C., Oksvold P., Mardinoglu A., Sivertsson A., Kampf C., Sjostedt E., Asplund A. (2015). Proteomics. Tissue-based map of the human proteome. Science.

[23] Alevizos I., Mahadevappa M., Zhang X., Ohyama H., Kohno Y., Posner M., Gallagher G.T., Varvares M., Cohen D., Kim D. (2001). Oral cancer in vivo gene expression profiling assisted by laser capture microdissection and microarray analysis. Oncogene.

[24] Yamashita K., Upadhyay S., Osada M., Hoque M.O., Xiao Y., Mori M., Sato F., Meltzer S.J., Sidransky D. (2002). Pharmacologic unmasking of epigenetically silenced tumor suppressor genes in esophageal squamous cell carcinoma. Cancer Cell.

[25] Tokumaru Y., Yamashita K., Osada M., Nomoto S., Sun D.I., Xiao Y., Hoque M.O., Westra W.H., Califano J.A., Sidransky D. (2004). Inverse correlation between cyclin A1 hypermethylation and p53 mutation in head and neck cancer identified by reversal of epigenetic silencing. Cancer Res..

[26] Wang L., Chen C., Li F., Hua Q.Q., Chen S.M., Xiao B.K., Dai M.Y., Li M., Zheng A.Y., Yu D. (2016). Overexpression of neuromedin U is correlated with regional metastasis of head and neck squamous cell carcinoma. Mol. Med. Rep..

[27] Garczyk S., Klotz N., Szczepanski S., Denecke B., Antonopoulos W., Von Stillfried S., Knchel R., Rose M., Dahl E. (2017). Oncogenic features of neuromedin U in breast cancer are associated with NMUR2 expression involving crosstalk with members of the WNT signaling pathway. Oncotarget.

[28] Rani S., Corcoran C., Shiels L., Germano S., Breslin S., Madden S., McDermott M.S., Browne B.C., O’Donovan N., Crown J. (2014). Neuromedin U: A Candidate Biomarker and Therapeutic Target to Predict and Overcome Resistance to HER-Tyrosine Kinase Inhibitors. Cancer Res..

[29] Harten S.K., Esteban M.A., Shukla D., Ashcroft M., Maxwell P.H. (2011). Inactivation of the von Hippel-Lindau tumour suppressor gene induces Neuromedin U expression in renal cancer cells. Mol. Cancer.

[30] Lin T.Y., Wu F.J., Chang C.L., Li Z.Y., Luo C.W. (2016). NMU signaling promotes endometrial cancer cell progression by modulating adhesion signaling. Oncotarget.

[31] Yang X.Y., Wang C.C., Lee W.Y.W., Trovik J., Chung T.K.H., Kwong J. (2018). Long non-coding RNA HAND2-AS1 inhibits invasion and metastasis in endometrioid endometrial carcinoma through inactivating neuromedin U. Cancer Lett..

[32] Ketterer K., Kong B., Frank D., Giese N.A., Bauer A., Hoheisel J., Korc M., Kleeff J., Michalski C.W., Friess H. (2009). Neuromedin U is overexpressed in pancreatic cancer and increases invasiveness via the hepatocyte growth factor c-Met pathway. Cancer Lett..

[33] Zhang S.L., Wang Q., Han Q., Han H.Z., Lu P.X. (2019). Identification and analysis of genes associated with papillary thyroid carcinoma by bioinformatics methods. Biosci. Rep..

[34] Shetzline S.E., Rallapalli R., Dowd K.J., Zou S.M., Nakata Y., Swider C.R., Kalota A., Choi J.K., Gewirtz A.M. (2004). Neuromedin U: A Myb-regulated autocrine growth factor for human myeloid leukemias. Blood.

[35] Harding M.A., Theodorescu D. (2007). RhoGDI2: A new metastasis suppressor gene: Discovery and clinical translation. Urol. Oncol. Semin. Orig. Investig..

[36] Wu Y., McRoberts K., Berr S.S., Frierson H.F., Conaway M., Theodorescu D. (2007). Neuromedin U is regulated by the metastasis suppressor RhoGDI2 and is a novel promoter of tumor formation, lung metastasis and cancer cachexia. Oncogene.

[37] Przygodzka P., Papiewska-Pajak I., Bogusz H., Kryczka J., Sobierajska K., Kowalska M.A., Boncela J. (2016). Neuromedin U is upregulated by Snail at early stages of EMT in HT29 colon cancer cells. Biochim. Biophys. Acta Gen. Subj..

[38] You S.J., Gao L. (2018). Identification of NMU as a potential gene conferring alectinib resistance in non-small cell lung cancer based on bioinformatics analyses. Gene.

[39] Martinez V.G., Crown J., Porter R.K., O’Driscoll L. (2017). Neuromedin U alters bioenergetics and expands the cancer stem cell phenotype in HER2-positive breast cancer. Int. J. Cancer.

[40] Mansoori B., Mohammadi A., Davudian S., Shirjang S., Baradaran B. (2017). The Different Mechanisms of Cancer Drug Resistance: A Brief Review. Adv. Pharm. Bull..

