# Erratum: Neuromedin U: A Small Peptide in the Big World of Cancer. *Cancers* 2019, *11*, 1312

**DOI:** 10.3390/cancers12010251

**Published:** 2020-01-20

**Authors:** Patrycja Przygodzka, Kamila Soboska, Ewelina Sochacka, Joanna Boncela

**Affiliations:** 1 Institute of Medical Biology, Polish Academy of Sciences, 106 Lodowa Str., 93-232 Lodz, Poland; ksoboska@cbm.pan.pl (K.S.); esochacka@cbm.pan.pl (E.S.); 2 Faculty of Biology and Environmental Protection, University of Lodz, 141/143 Pomorska Str, 90-236 Lodz, Poland

The authors wish to make the following corrections to this paper [1]:

The authors would like to replace references 23–40. The original references 23–40 are:

23. Alevizos, I.; Mahadevappa, M.; Zhang, X.; Ohyama, H.; Kohno, Y.; Posner, M.; Gallagher, G.T.; Varvares, M.; Cohen, D.; Kim, D.; et al. Oral cancer in vivo gene expression profiling assisted by laser capturemicrodissection and microarray analysis. *Oncogene* **2001**, *20*, 6196–6204.
24. Yamashita, K.; Upadhyay, S.; Osada, M.; Hoque, M.O.; Xiao, Y.; Mori, M.; Sato, F.; Meltzer, S.J.; Sidransky, D. Pharmacologic unmasking of epigenetically silenced tumor suppressor genes in esophageal squamous cell carcinoma. *Cancer Cell* **2002**, *2*, 485–495.
25. Tokumaru, Y.; Yamashita, K.; Osada, M.; Nomoto, S.; Sun, D.I.; Xiao, Y.; Hoque, M.O.; Westra, W.H.; Califano, J.A.; Sidransky, D. Inverse correlation between cyclin A1 hypermethylation and p53 mutation in head and neck cancer identified by reversal of epigenetic silencing. *Cancer Res.* **2004**, *64*, 5982–5987.
26. Wang, L.; Chen, C.; Li, F.; Hua, Q.Q.; Chen, S.M.; Xiao, B.K.; Dai, M.Y.; Li, M.; Zheng, A.Y.; Yu, D.; et al. Overexpression of neuromedin U is correlated with regional metastasis of head and neck squamous cell carcinoma. *Mol. Med. Rep.* **2016**, *14*, 1075–1082.
27. Garczyk, S.; Klotz, N.; Szczepanski, S.; Denecke, B.; Antonopoulos, W.; Von Stillfried, S.; Knchel, R.; Rose, M.;Dahl, E. Oncogenic features of neuromedin U in breast cancer are associated with NMUR2 expression involving crosstalk with members of theWNTsignaling pathway. *Oncotarget* **2017**, *8*, 36246–36265.
28. Rani, S.; Corcoran, C.; Shiels, L.; Germano, S.; Breslin, S.; Madden, S.; McDermott, M.S.; Browne, B.C.; O’Donovan, N.; Crown, J.; et al. Neuromedin U: A Candidate Biomarker and Therapeutic Target to Predict and Overcome Resistance to HER-Tyrosine Kinase Inhibitors. *Cancer Res.* **2014**, *74*, 3821–3833.
29. Harten, S.K.; Esteban, M.A.; Shukla, D.; Ashcroft, M.; Maxwell, P.H. Inactivation of the von Hippel-Lindau tumour suppressor gene induces Neuromedin U expression in renal cancer cells. *Mol. Cancer* **2011**, *10*.
30. Lin, T.Y.; Wu, F.J.; Chang, C.L.; Li, Z.Y.; Luo, C.W. NMU signaling promotes endometrial cancer cell progression by modulating adhesion signaling. *Oncotarget* **2016**, *7*, 10228–10242.
31. Yang, X.Y.; Wang, C.C.; Lee, W.Y.W.; Trovik, J.; Chung, T.K.H.; Kwong, J. Long non-coding RNA HAND2-AS1 inhibits invasion and metastasis in endometrioid endometrial carcinoma through inactivating neuromedin U. *Cancer Lett.* **2018**, *413*, 23–34.
32. Ketterer, K.; Kong, B.; Frank, D.; Giese, N.A.; Bauer, A.; Hoheisel, J.; Korc, M.; Klee, J.; Michalski, C.W.; Friess, H. Neuromedin U is overexpressed in pancreatic cancer and increases invasiveness via the hepatocyte growth factor c-Met pathway. *Cancer Lett.* **2009**, *277*, 72–81.
33. Zhang, S.L.; Wang, Q.; Han, Q.; Han, H.Z.; Lu, P.X. Identification and analysis of genes associated with papillary thyroid carcinoma by bioinformatics methods. *Biosci. Rep.* **2019**, *39*.
34. Shetzline, S.E.; Rallapalli, R.; Dowd, K.J.; Zou, S.M.; Nakata, Y.; Swider, C.R.; Kalota, A.; Choi, J.K.; Gewirtz, A.M. Neuromedin U: A Myb-regulated autocrine growth factor for human myeloid leukemias. *Blood* **2004**, *104*, 1833–1840.
35. Harding, M.A.; Theodorescu, D. RhoGDI2: A new metastasis suppressor gene: Discovery and clinical translation. *Urol. Oncol. Semin. Orig. Investig.* **2007**, *25*, 401–406.
36. Wu, Y.; McRoberts, K.; Berr, S.S.; Frierson, H.F.; Conaway, M.; Theodorescu, D. Neuromedin U is regulated by the metastasis suppressor RhoGDI2 and is a novel promoter of tumor formation, lung metastasis and cancer cachexia. *Oncogene* **2007**, *26*, 765–773.
37. Przygodzka, P.; Papiewska-Pajak, I.; Bogusz, H.; Kryczka, J.; Sobierajska, K.; Kowalska, M.A.; Boncela, J. Neuromedin U is upregulated by Snail at early stages of EMT in HT29 colon cancer cells. *Biochim. Biophys. Acta Gen. Subj.* **2016**, *1860*, 2445–2453.
38. You, S.J.; Gao, L. Identification of NMU as a potential gene conferring alectinib resistance in non-small cell lung cancer based on bioinformatics analyses. *Gene* **2018**, *678*, 137–142.
39. Martinez, V.G.; Crown, J.; Porter, R.K.; O’Driscoll, L. Neuromedin U alters bioenergetics and expands the cancer stem cell phenotype in HER2-positive breast cancer. *Int. J. Cancer* **2017**, *140*, 2771–2784.
40. Mansoori, B.; Mohammadi, A.; Davudian, S.; Shirjang, S.; Baradaran, B. The Different Mechanisms of Cancer Drug Resistance: A Brief Review. *Adv. Pharm. Bull*. **2017**, *7*, 339–348, doi:10.15171/apb.2017.041.

and should be changed to the following ones:

23. Yamashita, K.; Upadhyay, S.; Osada, M.; Hoque, M.O.; Xiao, Y.; Mori, M.; Sato, F.; Meltzer, S.J.; Sidransky, D. Pharmacologic unmasking of epigenetically silenced tumor suppressor genes in esophageal squamous cell carcinoma. *Cancer Cell* **2002**, *2*, 485–495, doi:10.1016/S1535-6108(02)00215-5.
24. Tokumaru, Y.; Yamashita, K.; Osada, M.; Nomoto, S.; Sun, D.I.; Xiao, Y.; Hoque, M.O.; Westra, W.H.; Califano, J.A.; Sidransky, D. Inverse correlation between cyclin A1 hypermethylation and p53 mutation in head and neck cancer identified by reversal of epigenetic silencing. *Cancer Res.* **2004**, *64*, 5982–5987, doi:Doi 10.1158/0008-5472.Can-04-0993.
25. Wang, L.; Chen, C.; Li, F.; Hua, Q.Q.; Chen, S.M.; Xiao, B.K.; Dai, M.Y.; Li, M.; Zheng, A.Y.; Yu, D.; et al. Overexpression of neuromedin U is correlated with regional metastasis of head and neck squamous cell carcinoma. *Mol. Med. Rep.* **2016**, *14*, 1075–1082, doi:10.3892/mmr.2016.5347.
26. Ketterer, K.; Kong, B.; Frank, D.; Giese, N.A.; Bauer, A.; Hoheisel, J.; Korc, M.; Kleeff, J.; Michalski, C.W.; Friess, H. Neuromedin U is overexpressed in pancreatic cancer and increases invasiveness via the hepatocyte growth factor c-Met pathway. *Cancer Lett.* **2009**, *277*, 72–81, doi:10.1016/j.canlet.2008.11.028.
27. Shetzline, S.E.; Rallapalli, R.; Dowd, K.J.; Zou, S.M.; Nakata, Y.; Swider, C.R.; Kalota, A.; Choi, J.K.; Gewirtz, A.M. Neuromedin U: A Myb-regulated autocrine growth factor for human myeloid leukemias. *Blood* **2004**, *104*, 1833–1840, doi:10.1182/blood-2003-10-3577.
28. Harding, M.A.; Theodorescu, D. RhoGDI2: A new metastasis suppressor gene: Discovery and clinical translation. *Urol. Oncol.-Semin. Ori.* **2007**, *25*, 401–406, doi:10.1016/j.urolonc.2007.05.006.
29. Wu, Y.; McRoberts, K.; Berr, S.S.; Frierson, H.F.; Conaway, M.; Theodorescu, D. Neuromedin U is regulated by the metastasis suppressor RhoGDI2 and is a novel promoter of tumor formation, lung metastasis and cancer cachexia. *Oncogene* **2007**, *26*, 765–773, doi:10.1038/sj.onc.1209835.
30. Przygodzka, P.; Papiewska-Pajak, I.; Bogusz, H.; Kryczka, J.; Sobierajska, K.; Kowalska, M.A.; Boncela, J. Neuromedin U is upregulated by Snail at early stages of EMT in HT29 colon cancer cells. *BBA-Gen. Subj.* **2016**, *1860*, 2445–2453, doi:10.1016/j.bbagen.2016.07.012.
31. You, S.J.; Gao, L. Identification of NMU as a potential gene conferring alectinib resistance in non-small cell lung cancer based on bioinformatics analyses. *Gene* **2018**, *678*, 137–142, doi:10.1016/j.gene.2018.08.032.
32. Lin, T.Y.; Wu, F.J.; Chang, C.L.; Li, Z.Y.; Luo, C.W. NMU signaling promotes endometrial cancer cell progression by modulating adhesion signaling. *Oncotarget* **2016**, *7*, 10228–10242, doi:10.18632/oncotarget.7169.
33. Yang, X.Y.; Wang, C.C.; Lee, W.Y.W.; Trovik, J.; Chung, T.K.H.; Kwong, J. Long non-coding RNA HAND2-AS1 inhibits invasion and metastasis in endometrioid endometrial carcinoma through inactivating neuromedin U. *Cancer Lett.* **2018**, *413*, 23–34, doi:10.1016/j.canlet.2017.10.028.
34. Garczyk, S.; Klotz, N.; Szczepanski, S.; Denecke, B.; Antonopoulos, W.; Von Stillfried, S.; Knchel, R.; Rose, M.; Dahl, E. Oncogenic features of neuromedin U in breast cancer are associated with NMUR2 expression involving crosstalk with members of the WNT signaling pathway. *Oncotarget* **2017**, *8*, 36246–36265, doi:10.18632/oncotarget.16121.
35. Martinez, V.G.; Crown, J.; Porter, R.K.; O’Driscoll, L. Neuromedin U alters bioenergetics and expands the cancer stem cell phenotype in HER2-positive breast cancer. *Int. J. Cancer* **2017**, *140*, 2771–2784, doi:10.1002/ijc.30705.
36. Rani, S.; Corcoran, C.; Shiels, L.; Germano, S.; Breslin, S.; Madden, S.; McDermott, M.S.; Browne, B.C.; O’Donovan, N.; Crown, J.; et al. Neuromedin U: A Candidate Biomarker and Therapeutic Target to Predict and Overcome Resistance to HER-Tyrosine Kinase Inhibitors. *Cancer Res.* **2014**, *74*, 3821–3833, doi:10.1158/0008-5472.Can-13-2053.
37. Harten, S.K.; Esteban, M.A.; Shukla, D.; Ashcroft, M.; Maxwell, P.H. Inactivation of the von Hippel-Lindau tumour suppressor gene induces Neuromedin U expression in renal cancer cells. *Mol. Cancer* **2011**, *10*, 89.
38. Zhang, S.L.; Wang, Q.; Han, Q.; Han, H.Z.; Lu, P.X. Identification and analysis of genes associated with papillary thyroid carcinoma by bioinformatics methods. *Bioscience Rep.* **2019**, *39*, doi:10.1042/Bsr20190083.
39. Alevizos, I.; Mahadevappa, M.; Zhang, X.; Ohyama, H.; Kohno, Y.; Posner, M.; Gallagher, G.T.; Varvares, M.; Cohen, D.; Kim, D.; et al. Oral cancer in vivo gene expression profiling assisted by laser capture microdissection and microarray analysis. *Oncogene* **2001**, *20*, 6196–6204, doi:10.1038/sj.onc.1204685.
40. Mansoori, B.; Mohammadi, A.; Davudian, S.; Shirjang, S.; Baradaran, B. The Different Mechanisms of Cancer Drug Resistance: A Brief Review. *Adv. Pharm. Bull.* **2017**, *7*, 339–348, doi:10.15171/apb.2017.041.

The Editorial Team would like to apologize for any inconvenience caused to the readers by these changes.
